# Characterization of spleen and lymph node cell types *via* CITE-seq and machine learning methods

**DOI:** 10.3389/fnmol.2022.1033159

**Published:** 2022-10-13

**Authors:** Hao Li, Deling Wang, Xianchao Zhou, Shijian Ding, Wei Guo, Shiqi Zhang, Zhandong Li, Tao Huang, Yu-Dong Cai

**Affiliations:** ^1^College of Biological and Food Engineering, Jilin Engineering Normal University, Changchun, China; ^2^State Key Laboratory of Oncology in South China, Department of Radiology, Collaborative Innovation Center for Cancer Medicine, Sun Yat-sen University Cancer Center, Guangzhou, China; ^3^Center for Single-Cell Omics, School of Public Health, Shanghai Jiao Tong University School of Medicine, Shanghai, China; ^4^School of Life Sciences, Shanghai University, Shanghai, China; ^5^Key Laboratory of Stem Cell Biology, Shanghai Institutes for Biological Sciences (SIBS), Shanghai Jiao Tong University School of Medicine (SJTUSM), Chinese Academy of Sciences (CAS), Shanghai, China; ^6^Department of Biostatistics, University of Copenhagen, Copenhagen, Denmark; ^7^CAS Key Laboratory of Computational Biology, Bio-Med Big Data Center, Shanghai Institute of Nutrition and Health, University of Chinese Academy of Sciences, Chinese Academy of Sciences, Shanghai, China; ^8^CAS Key Laboratory of Tissue Microenvironment and Tumor, Shanghai Institute of Nutrition and Health, University of Chinese Academy of Sciences, Chinese Academy of Sciences, Shanghai, China

**Keywords:** spleen and lymph, machine learning algorithm, feature analysis, deep forest, decision rule

## Abstract

The spleen and lymph nodes are important functional organs for human immune system. The identification of cell types for spleen and lymph nodes is helpful for understanding the mechanism of immune system. However, the cell types of spleen and lymph are highly diverse in the human body. Therefore, in this study, we employed a series of machine learning algorithms to computationally analyze the cell types of spleen and lymph based on single-cell CITE-seq sequencing data. A total of 28,211 cell data (training vs. test = 14,435 vs. 13,776) involving 24 cell types were collected for this study. For the training dataset, it was analyzed by Boruta and minimum redundancy maximum relevance (mRMR) one by one, resulting in an mRMR feature list. This list was fed into the incremental feature selection (IFS) method, incorporating four classification algorithms (deep forest, random forest, K-nearest neighbor, and decision tree). Some essential features were discovered and the deep forest with its optimal features achieved the best performance. A group of related proteins (CD4, TCRb, CD103, CD43, and CD23) and genes (Nkg7 and Thy1) contributing to the classification of spleen and lymph nodes cell types were analyzed. Furthermore, the classification rules yielded by decision tree were also provided and analyzed. Above findings may provide helpful information for deepening our understanding on the diversity of cell types.

## Introduction

The spleen and lymph nodes are both important secondary lymphoid organs in the human body for immune response and other immunological functions. The human body has hundreds of lymph nodes, which are strategically distributed throughout the body for lymphocytes to effectively encounter antigens and be activated (Koning and Mebius, 2012). When the body is stimulated by foreign substances, immune cells recognize foreign antigens and bring them back to the lymph nodes and recirculate through the lymph nodes. Thus, a few antigen-specific lymphocyte populations are continuously transported, resulting in immune cells in the lymph nodes throughout the body to respond (Girard et al., 2012). This mechanism forms the body’s systemic immune surveillance in response to foreign invasions or alterations in the body’s own cells. The spleen is the largest secondary lymphoid organ in the body, and its functions include blood filtration, hematopoiesis, red blood cell clearance, and various important immune functions (Lewis et al., 2019). The spleen can be divided into two components in structure, including the red pulp, which is responsible for blood filtration function, and the white pulp, which is filled with lymphoid cells (Mebius and Kraal, 2005).

Different immune cells are present in the lymph nodes, and they are all essential for the lymph node functions. During immune surveillance, immune cells need to be continuously transported in and out of the lymph nodes to ensure contact with the foreign invaders and transport the antigen back to the lymph node. Immune cells enter the lymph nodes mainly through high endothelial venules and afferent lymphatics and flow out through the efferent lymphatics (von Andrian and Mempel, 2003). Within the lymph node, various immune cells have clear spatial positioning and movement based on the fibroblast reticulum cells networks, and under the guidance of the network structure, soluble antigen and cytokines can be delivered *via* the lymph conduit and further provide conditions for the survival and activation of various immune cells (Chang and Turley, 2015). During immune response, the resident dendritic cells integrated in the lymphatic sinuses actively recognize antigens from the lymph fluid and quickly present the antigens to CD4+ T cells and CD8+ T cells after being stimulated by the antigens (Gerner et al., 2015). Moreover, the antigen acquisition of B cell depend on follicular dendritic cells and macrophage (Batista and Harwood, 2009).

Immune cells in the spleen also play an important role. The B cells in the spleen have a series of distinct B cell lineages. In addition to traditional and mature B cells, the functions of marginal zone (MZ) B cells, B1 B cell, and follicular B cells are very important. MZ B cells reside between the MZ and red pulp, and it is an innate-like B cell subset specific to the spleen. It can quickly produce IgM and class-switched IgG and IgA antibodies against common antigens in the first-line defense (Cerutti et al., 2013). MZ B cell can also activate NKT cells through surface CD1d molecules and promote NKT cells to produce inflammatory cytokines (Semmling et al., 2010). B1 B cell is similar to MZ B cell, produces IgM antibodies, and contributes to circulating “natural” antibodies. In addition, the antibody production is independent of thymus cells, and B1 B cell can participate in memory responses (Baumgarth, 2011; Cerutti et al., 2013). Follicular B cells circulate between the blood and the spleen and respond to antigens through thymus-dependent signals (Hoek et al., 2010). Monocytes have two main functions, namely, circulating in the system and mobilizing to the tissues when needed, and monitoring foreign infections in the blood. It can also differentiate into various myeloid cell types, such as DC and macrophages (Geissmann et al., 2010; Lewis et al., 2019). T cells, together with B cells, are present throughout the spleen, and they ear pivotal in mediating adaptive immunity. During an active immune response, T and B cells achieve different functions and localization changes through surface receptor expression and chemotactic gradients (Pereira et al., 2010; Eisenbarth, 2019). In brief, the function of immune cells in the spleen and lymph nodes is very important, and it is an indispensable line of defense for the human body against foreign invasion and changes in its cells.

Advances in single-cell sequencing, such as the CITE-seq method (measurement with cellular indexing of transcriptomes and epitopes by sequencing) (Stoeckius et al., 2017), have provided precise tools for the comprehensive analysis of the immune system. CITE-seq method can be utilized for the analysis of immune cells at single-cell resolution, determination of the interaction between different immune cell groups, and identification of novel distinct immune cell subsets in health and disease (Papalexi and Satija, 2018). For example, the single-cell study of mouse and human lymph nodes has clarified the diversity of cell populations and the underlying developmental and structural organization (Xiang et al., 2020). Single-cell sequencing can also discover cell subgroups that contribute to the disease. For example, splenic single-cell sequencing of rituximab therapy in patients with immune thrombocytopenia found that some specific B cell subgroups are associated with patient recurrence; and this type of B cell subgroup comprised highly expressed CD19 molecules on the surface, and these molecules may be potential therapeutic targets (Crickx et al., 2021).

In the present study, based on the CITE-seq sequencing data of immune cells from murine spleen and lymph nodes, we computationally analyzed such data to extract gene expression signatures and biomarkers, which can characterize different spleen and lymph node cell types. Briefly, the Boruta (Kursa and Rudnicki, 2010) and minimum redundancy and maximum relevance (mRMR) (Peng et al., 2005) methods were applied on the training dataset one by one. The mRMR feature list was obtained, which was further introduced to the incremental feature selection (IFS) (Liu and Setiono, 1998). Four classification algorithms, including deep forest (DF) (Zhou and Feng, 2018), random forest (RF) (Breiman, 2001), K-nearest-neighbor (KNN) (Cover and Hart, 1967), and decision tree (DT) (Safavian and Landgrebe, 1991) were attempted in the IFS method. Through above procedures, gene-expression/protein-abundance features and decision rules were obtained, which were essential for distinguishing 24 spleen and lymph node cell types. These proteins, genes and rules provided a much clearer view of the relationship among transcriptional variation, cell phenotype, and function of different cell subtypes. Some new expression patterns, implied by rules, may also reveal new cell markers or functions that have not been determined. Furthermore, the optimal classifiers were also constructed through IFS method, which were further evaluated on the test dataset.

## Materials and methods

### Study design

**FIGURE 1:**
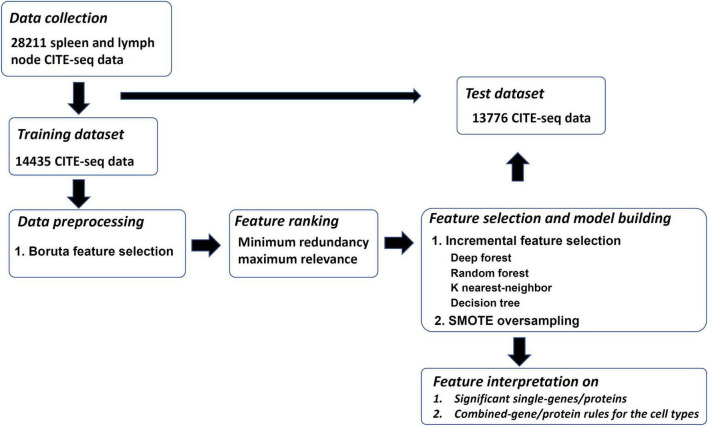
Overview of the design. The training and test datasets for spleen and lymph are investigated. The training dataset is analyzed by the feature selection methods of Boruta and minimum redundancy maximum relevance one by one. The mRMR feature list is generated, which is further fed into incremental feature selection (IFS) method, incorporating four classification algorithms, to extract significant single-genes/proteins and combined-gene/protein rules. The SMOTE is applied in the IFS method to reduce the influence of imbalanced problem. The optimal classifiers yielded by the IFS method are applied on the test dataset to evaluate their generalization ability.

Our research is divided into the following processes: (1) data collection, (2) classification into training and test datasets, (3) pre-processing of data, (4) feature ranking, (5) development of models, and (6) feature interpretation, as shown in Figure 1. The details of the whole process are described in the following sections.

### Data collection

We downloaded the single-cell CITE-seq protein and gene expression data of spleen and lymph from Gayoso et al. (2021) at https://github.com/YosefLab/totalVI_reproducibility/blob/v0.3/data/spleen_lymph_111.h5ad and https://github.com/YosefLab/totalVI_reproducibility/blob/v0.3/data/spleen_lymph_206.h5ad. To refine these two datasets, we only selected the 110 proteins and 13,553 genes that overlapped and the cells from the 24 overlapped cell types. After filtering, the first dataset included 14,435 cells, which was picked up as the training dataset, and the second dataset, including 13,776 cells, was termed as the test dataset. The sample sizes of each cell type in the training and test datasets are listed in Table 1.

**TABLE 1:** The sample sizes of each cell type in the training and test datasets.

Index	Cell type	Sample size of the training dataset	Sample size of the test dataset
1	Lymph node CD122+ CD8 T	551	551
2	Lymph node CD4 T	1,777	1,761
3	Lymph node CD8 T	1,031	1,046
4	Lymph node Ifit3-high B	404	375
5	Lymph node Ifit3-high CD4 T	267	256
6	Lymph node Ifit3-high CD8 T	160	161
7	Lymph node mature B	1,953	1,917
8	Lymph node transitional B	102	103
9	Lymph node tregs	171	171
10	Spleen B1 B	294	270
11	Spleen CD122+ CD8 T	267	261
12	Spleen CD4 T	818	818
13	Spleen CD8 T	427	421
14	Spleen cDC2s	119	122
15	Spleen cycling B/T cells	123	119
16	Spleen ICOS-high tregs	117	116
17	Spleen Ifit3-high B	273	249
18	Spleen Ly6-high mono	129	114
19	Spleen mature B	3,187	2,873
20	Spleen MZ B	431	458
21	Spleen neutrophils	159	144
22	Spleen NK	155	130
23	Spleen NKT	176	155
24	Spleen transitional B	1,344	1,185
	Total	14,435	13,776

The download data were all CITE-sequencing data. CITE-seq method is an effective tool for single-cell level multi-omics integrative analyses and were used to identify transcriptomics profiling together with phenotypic or proteomic signatures. The general workflow of CITE-seq can be divided into three steps as follows:

1) Antibodies with specific DNA barcodes can specifically bind to the target proteins/antigens;
2) By using single-cell droplet library preparation methods, such as the preparation methods of 10X genomics, drop-seq, and ddseq, each cell together with its unique DNA barcodes was isolated in one droplet; and
3) All the mRNAs of this single cell are indexed with the original DNA barcodes. Finally, by using next-generation sequencing and proper bioinformatics methods, we obtained the single-cell mRNA profiling information together with its respective phenotypes, such as biomarker patterns.

### Data preprocessing

Lots of features (110 proteins and 13,553 genes) were used to represent cells in the training and test datasets. Clearly, it was impossible that all of them were related to distinguish cell types. Generally, the related features occupy a small proportion. Thus, it was necessary to extract them with some advanced computational methods. Here, we selected Boruta feature selection method (Kursa and Rudnicki, 2010). Its brief description was as follows.

Boruta feature selection method is a RF (Breiman, 2001)-based wrapper method that is used to detect and output relevant features. The importance of one feature is measured by comparing it with shuffled features. For each real feature in the original dataset, a shuffled feature is generated. All shuffled and real features are combined to constitute a shuffled dataset. A RF classifier is trained on such shuffled dataset to produce the importance score of each feature. The real features with importance scores significantly higher than those for shuffled features are kept. These features are removed from the dataset and the same procedures are applied to the updated dataset until the time reaches a predefined value. The Boruta outputs all kept features, which are deemed to be important.

In the present study, the Boruta program provided by https://github.com/scikit-learn-contrib/boruta_py was applied, and the default parameters were set.

### Feature ranking

The features selected by Boruta should be further evaluated as it was not clear which features were more important. Thus, we further employed the widely used feature selection method, mRMR (Peng et al., 2005).

The mRMR is a high-performance feature selection method. The original purpose was to select a compact feature subset that have high relevance to target labels and low redundancies between features in this set. However, such purpose is hard to be achieved as such problem is NP-hard. As an alternative way, it sorts features in a list, named mRMR feature list. Features with high ranks in this list are more important than others. This list is generated by repeatedly selecting features. In each loop, a feature with maximum difference between its relevance to target labels and redundancies to already-selected features is selected and appended to the current list. The relevance and redundancy are all measured by mutual information (MI) between features or target labels.

In the present study, the mRMR program with the default parameters was obtained from http://home.penglab.com/proj/mRMR/.

### Feature selection and model building

Although mRMR generated the mRMR feature list, it was still not clear which features can constitute the optimal features for distinguishing cell types. In view of this, some other computational methods were adopted.

#### Incremental feature selection

IFS is a feature selection method (Liu and Setiono, 1998), which can determine the optimal features for a given classification algorithm. Based on one feature list (e.g., mRMR feature list), a series of feature subsets are generated by IFS, each of which contains some top features in the list. For example, the first subset can contain the first feature in the list, the second subset includes the first two features in the list, and so forth. On each feature subset, a specific classifier is constructed on samples represented by features in this subset with a given classification algorithm. All classifiers are further evaluated by 10-fold cross-validation (Kohavi, 1995). The classifier with the best performance is regarded as the optimal classifier, and the features used in the optimal classifier are regarded as the optimal features.

#### Classification algorithm

To execute the IFS method, at least one classification algorithm is necessary. To fully evaluate each feature subset, four powerful classification algorithms were attempted, including DF (Zhou and Feng, 2018), RF (Breiman, 2001), KNN (Cover and Hart, 1967), and DT (Safavian and Landgrebe, 1991).

##### Deep forest

DF is a deep neural network that builds deep models based on non-differentiable modules, such as DTs. Briefly, DF builds multi-layer models through tree integration by using gradient boosting DTs as a building block for each layer, emphasizing its representational learning capability and optimization of the training process. The DF algorithm used in the present study was the Cascaded DF (El-Nabawy et al., 2021), which can be trained to obtain hierarchical and distributed representations in both supervised and unsupervised settings. This structure can achieve competitive performance on many types of tasks. It contains a smaller number of parameters, ensuring that a large number of tuning parameters is not required during use, improving training efficiency.

##### Random forest

The RF is a non-parametric decision classification ensemble algorithm containing a number of DTs. Each DT is constructed by randomly selecting samples and features. RF aggregates the predictions of all DTs as its decision. Considering the differences between DTs, RF can avoid the overfitting problem. Although RF loses the interpretability and may slightly increase the bias, it improves the performance and reduces the error. To date, it is always an important candidate to construct classifiers in dealing with different medical problems (Casanova et al., 2014; Marques et al., 2016; Zhao et al., 2018; Baranwal et al., 2019; Chen W. et al., 2021; Chen et al., 2022; Ran et al., 2022; Tang and Chen, 2022; Wang and Chen, 2022; Wu and Chen, 2022; Yang and Chen, 2022).

##### K-nearest-neighbor

The KNN is one of the most classic classification algorithms. This algorithm does not contain a training procedure. For a given test sample, its distances to all training samples are calculated and *k* nearest neighbors are extracted. Based on the labels of these neighbors, the predicted label of the test sample can be determined. Generally, the label occurring most on the *k* nearest neighbors is picked up as the predicted label of the test sample.

##### Decision tree

DT is also a classic classification algorithm. Different from above algorithms, which is very difficult to uncover their classification principles, DT provides an opportunity for human to understand its classification procedures as such procedures are completely open. Besides the tree-like representation of DT, it can also be represented by a set of rules. Each rule indicates a path from the root to one leaf node. Several features may be included in each rule, which constitute a special pattern for the result of the rule (label for one class). In recent years, it becomes more and more popular to analyze various complicated medical datasets (Chen L. et al., 2021; Ding et al., 2022; Zhou et al., 2022).

To implement above four classification algorithms, some public online sources were used in this study. In detail, the DF program was downloaded from https://github.com/LAMDA-NJU/Deep-Forest. As for RF, KNN, and DT, their programs implemented through the Scikit-learn module were used. All programs were executed using default parameters.

#### Synthetic minority oversampling technique

According to Table 1, the training dataset was imbalanced. The size of the largest cell type was about 31 times the size of the smallest cell type. The classifiers directly built on such dataset may produce bias. This problem can be completely or partly solved by some computer techniques. Here, we adopted synthetic minority oversampling technique (SMOTE) (Chawla et al., 2002). It creates synthetic samples for each minority class until the size of the minority class is equal to that of the majority class. The SMOTE program used in this study was sourced from https://github.com/scikit-learn-contrib/imbalanced-learn and it was performed with the default parameters.

### Feature interpretation

The feature interpretation includes single-genes/proteins and combined gene/protein rules. The interpretation of single genes/proteins focuses on the essential genes/proteins that are assigned high ranks in the mRMR feature list, while that of the combined gene/protein rule focuses on the rules yielded by the optimal DT classifier.

### Performance evaluation

The Matthew correlation coefficient (MCC) (Matthews, 1975) metric is used to evaluate the performance of all classifiers. MCC is the correlation coefficient between the observed labels and the predicted labels. It is deemed as a balanced measurement even if the sample sizes of classes differ significantly. MCC can be calculated as follows:


(1) $$\begin{array}{l} \text{MCC }\;=\;\frac{\text{cov}(\text{X},\text{Y})}{\sqrt{\text{cov}\left(\text{X},\text{X}\right)\text{cov}(\text{Y},\text{Y})}}\;\\ \;\;\;\;=\;\frac{\sum_{\text{i}=1}^{\text{n}}\;\sum_{\text{j}=1}^{\text{C}}\left({\text{x}}_{\text{ij}}-{\bar{\text{x}}}_{\text{j}}{\text{)(y}}_{\text{ij}}-{\bar{\text{y}}}_{\text{j}}\right)}{\sqrt{\sum_{\text{i}=1}^{\text{n}}\;\sum_{\text{j}=1}^{\text{c}}\;{({\text{x}}_{\text{ij}}-{\bar{\text{x}}}_{\text{j}}\text{)}}^{2}\sum_{\text{i}=1}^{\text{n}}\sum_{\text{j}=1}^{\text{c}}{{\text{(y}}_{\text{ij}}-{\bar{\text{y}}}_{\text{j}})}^{2}}}, \end{array}$$

where *X* denotes a binary matrix including the predicted label of each sample, and *Y* denotes another binary matrix representing the true label of each sample. The correlation coefficient of *X* and *Y* is defined as cov (*X, Y*), and ${\bar{x}}_{j}$ and ${\bar{y}}_{j}$ are the average values in the jth column of *X* and *Y*, respectively. *C* denotes the total number of cell types, while *n* denotes the number of cells. The range of MCC is between −1 and 1. Higher values of MCC indicate better performance of the classifier.

In addition, we also provided the overall accuracy and individual accuracy on each cell type to fully display the performance of all classifiers.

## Results

In the present study, 28,211 cells were obtained from the two sample sets. After filtration, the two datasets, including 14,435 and 13,776 cells, respectively, were deeply analyzed. The entire procedures are illustrated in Figure 1. This section gave the detailed results at each stage.

### Prediction performance

As lots of features were involved to represent cells, we first adopted Boruta to select important features from the training dataset. 1,180 features were kept, where 67 were about proteins and rest 1,113 were about genes. These remaining features were further analyzed by the mRMR method. An mRMR feature list was generated, which is provided in Supplementary Table 1.

**FIGURE 2:**
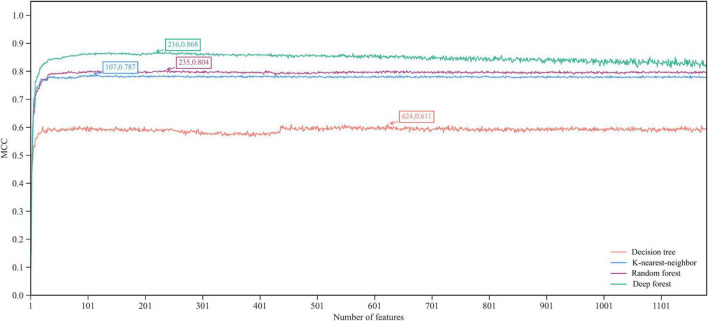
The IFS curves of four classification algorithms in terms of MCC. The highest MCC values of four algorithms are marked. The deep forest yields the highest MCC of 0.868 when top 216 features in the mRMR feature list are used.

**FIGURE 3:**
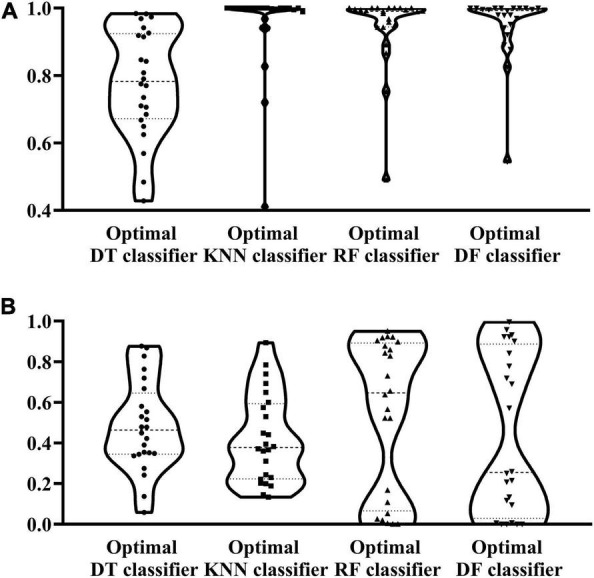
Violin plot to show the performance of four optimal classifiers on the training and test datasets. **(A)** Violin plot on the training dataset. **(B)** Violin plot on the test dataset.

According to the procedures shown in Figure 1, the IFS method was applied to the mRMR feature list. We selected step one to construct all possible feature subsets from such list, obtaining 1,180 feature subsets. On each subset, a classifier was built with each of four classification algorithms (DF, RF, KNN, and DT) and cells represented by features in this subset. All classifiers were evaluated by 10-fold cross-validation. Their performance was assessed by measurements listed in section “Performance evaluation,” which is available in Supplementary Table 2. To clearly show the performance of classifiers under different feature subsets, an IFS curve was plotted for each classification algorithm, as shown in Figure 2, in which MCC was set as Y-axis and the size of the feature subset (i.e., the number of features) was defined as X-axis. It can be observed that when using DF as the classification algorithm, the highest level of MCC of 0.868 was obtained at the top 216 features. The highest level of MCC of 0.804 can be achieved at the top 235 features by using RF, whereas the highest levels of MCC of 0.787 and 0.611 can be obtained at the top 107 and 624 features by using KNN and DT, respectively. Accordingly, the optimal features for each classification algorithm can be obtained and the optimal DF, RF, KNN, and DT classifiers can be built with their optimal features. The ACC values for these classifiers are listed in Table 2, which were 0.881, 0.819, 0.799, and 0.643, respectively. From ACC and MCC, the optimal DF classifier was best, the optimal RF and KNN classifiers were almost at the same level, and the optimal DT classifier provided the lowest performance. Furthermore, we also counted the performance of these four optimal classifiers on 24 cell types. A violin plot was drawn for each optimal classifier, which is displayed in Figure 3A. Evidently, the optimal DT classifier still gave the lowest performance. As for other three optimal classifiers, the optimal KNN classifier seemed to be better than the optimal RF and DF classifiers. However, its performance on majority class was very poor. For example, on the largest cell type (Spleen Mature B), the optimal KNN classifier only provided the individual accuracy of 0.410. Such case decreased its overall performance. Furthermore, the optimal DF classifier was better than the optimal RF classifier based on their performance on 24 cell types.

**TABLE 2:** Performance of the optimal classifiers with different classification algorithms on the training and test datasets.

Classification algorithm	Number of features	Training dataset		Test dataset	
			
		ACC	MCC	ACC	MCC
Deep forest	216	0.881	0.868	0.586	0.542
Random forest	235	0.819	0.804	0.599	0.552
K-nearest-neighbor	107	0.799	0.787	0.361	0.322
Decision tree	624	0.643	0.611	0.450	0.395

To test the generalization ability of above four optimal classifiers, the test dataset was fed into each optimal classifier. The obtained ACC and MCC values are listed in Table 2. It can be observed that the ACC and MCC values on testing dataset were lower than those on the training dataset. As testing samples did not participate in the construction of classifiers, such results were acceptable. In detail, the optimal DF and RF classifier yielded the most robust prediction with MCC values of 0.542 and 0.552, respectively, and the ACC values of 0.586 and 0.599, respectively. The MCC and ACC values of the optimal KNN and DT classifiers are much lower than those obtained by the above two classifiers. As for their performance on 24 cell types, a violin plot was also drawn for each optimal classifier, as illustrated in Figure 3B, from which we can see that the high individual accuracies of the optimal RF and DF classifiers were evidently more than those of other two optimal classifiers, conforming to their overall performance.

### Significant single-genes/proteins and combined gene/protein rules

**FIGURE 4:**
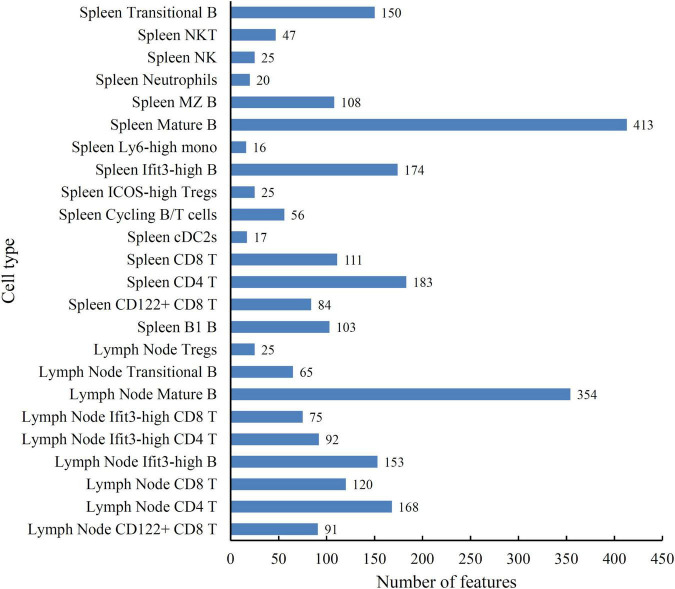
Bat chart to show the number of rules for each cell type.

According to the principle for generating the mRMR feature list, top features in such list may be essential for distinguishing cell types. The discussion part in section “Analysis of the quantitative features for distinguishing different cell types” shown that the surface proteins (CD4, TCRb, CD103, CD43, and CD23) and genes (Nkg7 and Thy1) have a decisive effect on different cell types. However, considering that the interaction between features is unknown, relying on one single protein or gene alone to reveal the different expression patterns of spleen and lymph node cells is not sufficient. Therefore, we further employed DT to learn classification rules. As the optimal DT classifier used the top 624 features in the mRMR feature list, a tree was learn by applying DT on all samples represented by these features. From this tree, a total of 2,675 rules were generated, which are given in Supplementary Table 3. Each cell type was assigned some rules. The number of rules for each cell type is shown in Figure 4. The cell type (Spleen Mature B) was assigned most rules, whereas the rules for the cell type (Spleen Ly6-high mono) were least. In section “Analysis of the quantitative rules for distinguishing different cell types,” some rules were analyzed.

## Discussion

As mentioned above, we utilized several machine learning methods to characterize the relationship among cell phenotypes, functions, and transcriptomes by analyzing CITE-seq data of immune cells from murine spleen and lymph nodes. The features in the investigated datasets contain the abundance of 110 surface proteins and the expression level of 13,553 genes. After using several methods, we finally constructed a DF classifier using 216 features, which can yield a high MCC of 0.868. Some top features in the mRMR feature list were deemed to be essential to distinguish spleen and lymph node cells. In addition, we further adopted DT to generate a group of decision rules, which can not only distinguish cell types but also indicate different patterns on various cell types.

### Analysis of the quantitative features for distinguishing different cell types

Based on machine learning algorithms, we identified a set of significant features that contribute to the determination of immune cell types in the spleen and lymph nodes. We reviewed several publications to prove how these features are decisive for different cell types, including surface proteins of CD4, TCRb, CD103, CD43, and CD23, and the genes of Nkg7 and Thy, which are listed in Table 3.

**TABLE 3:** Essential features for identifying spleen and lymph node cell types.

Feature name	Related proteins/genes	Rank in the mRMR feature list
ADT_CD4_A0001	CD4	1
ADT_TCRbchain_A0120	TCRb	5
ADT_CD103_A0201	CD103	12
ADT_CD43_A0110	CD43	14
ENSMUSG00000004612	Nkg7	15
ENSMUSG00000032011	Thy1	16
ADT_CD23_A0108	CD23	28

The surface protein CD4 (ADT_CD4_A0001) is a coreceptor of T cell receptors on T lymphocytes, and it can recognize antigens displayed by MHC class II molecules on antigen presenting cells. CD4 is a commonly used marker for CD4+ T cells. CD4 + T cells can differentiate into various effector cell subtypes, including T types 1, 2, and 17, follicular helper T cells, and regulatory T cells. These subtypes are all involved in regulating the immune response to different types of pathogens (Hwang et al., 2020). Similarly, the CD8a (ADT_CD8a_A0002) and CD8b (ADT_CD8b (Ly-3)_A0230) proteins are surface glycoproteins found on most cytotoxic T lymphocytes. They act as a coreceptor of T cell receptors and can recognize antigens in an MHC class I context by antigen presenting cells. During T cell differentiation in the thymus, precursors cells developed from CD4 CD8 double negative cells into CD4 CD8 double positive cells. Then, these double positive cells make a lineage decision to become CD4+ T cell or CD8+ T cells and retain the specificity to MHC class II or MHC class I (Germain, 2002). Therefore, the expression level of Cd8b1 (ENSMUSG00000053044) and Cd8a (ENSMUSG00000053977) are also important for distinguishing T cells in the transcriptome level.

The surface protein TCRb (ADT_TCRbchain_A0120) is a subunit of the T cell receptor molecules. It can be used as a marker of T cells and is important for TCR-mediated T cell recognition of foreign antigens displayed by MHC. At the DNA level, the TCRB is synthesized through VDJ recombination events and later connected to the C segment at the RNA level. These events play an important role in the development of T cells and can provide a wide range of antigen recognition for immune cells (Roldan et al., 1995).

Protein CD103 (ADT_CD103_A0201) is also known as integrin subunit alpha E. It is highly expressed in intestinal intraepithelial lymphocytes and could be used as a biomarker for CD4+ T cells. CD103+ CD8+ T cells induced by human alloantigens have the functional characteristics of regulatory T cells, thus confirming the importance of CD103 to CD4+ T cells, especially regulatory T cells (Uss et al., 2006). The negative expression of CD103 is associated with splenic marginal zone lymphoma and mantle cell lymphoma, and the pathogenic mechanism may be related to the change in interaction between CD103 and E-cadherin (Santos et al., 2017).

The surface protein CD43 (ADT_CD43_A0110) is a highly sialylated transmembrane glycoprotein, and it participates in cell adhesion, proliferation and the antigen-specific activation of T cells. It is expressed in various immune cells and can be used as a marker for NK cells and B cells (McCann et al., 2003). High CD43 level is related to diffuse large B cell lymphoma, and CD43 could be used as a biomarker of adverse prognosis (Ma et al., 2015).

Gene Nkg7 (ENSMUSG00000004612) encodes a protein called natural killer cell granule protein 7, which is expressed in activated T cells and natural killer cells but not in B cells, monocytes, and myeloid cells (Turman et al., 1993). A recent study found that Nkg7 was up-regulated dozens of times during the differentiation of NKT cell lineages, indicating its important role in the activity of NKT cells. Moreover, experimental results by using Nkg7 knockout mice show that the negative expression of Nkg7 would decrease NK cell-mediated cytotoxicity (Baxter et al., 2016). Therefore, Nkg7 is essential for the functions of T, NK, and NKT and can be used as an important feature for distinguishing different immune cells.

Gene Thy1 (ENSMUSG00000032011) encodes a cell surface glycoprotein that belongs to the immunoglobulin superfamily. It was first identified in mouse T lymphocytes and was later shown to play a role in cell adhesion and communication of multiple cell types (Sauzay et al., 2019). Moreover, Thy1 is expressed in B cells in the bone, marrow, spleen, and lymph node, and has different expression patterns (Crawford and Goldschneider, 1980). This differential expression of Thy1 can be used to identify B cells in different tissues.

The protein CD23 (ADT_CD23_A0108) is a B cell-specific antigen that plays an essential role in B cell growth and differentiation and the regulation of IgE production. The stimulation of CD40 antigen induces the expression of CD23 and participates in the activation and maturation of B cells, and CD23 could be used as a marker of mature B cells (Saeland et al., 1993).

The features generated by the machine learning algorithms are closely related to the cell function or fate of immune cells. The differential expression of multiple genes in different cell types or tissues provides a basis for our classifier.

### Analysis of the quantitative rules for distinguishing different cell types

Our results show a large number of decision rules (Supplementary Table 3). In some decision rules, the expression of some commonly used markers provides proof of reliability of the rules. Moreover, some surface protein abundance and gene expression levels may show new characteristics of cell types and may reveal the relationship among transcriptional variation, cell phenotype, and function. The obtained decision rules involved the surface proteins of CD24, CD103, CD357, CD43, and so on.

In our decision rules for indicating spleen mature B cells, we noticed a criterion that requires the high expression of surface protein CD24. CD24 is commonly expressed on mature granulocytes and B cells, and it is very crucial for the differentiation process of cells (Pruszak et al., 2009). CD24 is a crucial signal molecule that is expressed on human B cells and participates in the activation of B cell functions (Kay et al., 1991). The highly expressed CD24 on B cells is associated with several diseases, including small cell lung carcinoma and systemic lupus erythematosus (Jackson et al., 1992; Jin et al., 2013). The expression pattern of CD24 can serve as the indicator for identifying the B cell maturation stages, and the highly presented CD24 antigen on B cells is precisely related to the maturation of B cells (Lavabre-Bertrand et al., 1994). These findings are consistent with our decision rules for indicating spleen mature B cells, which support the reliability of our analysis.

The surface protein CD103, which is also called ITGAE, showed a high expression for indicating lymph node CD8 T cells based on our analysis. This protein is preferentially expressed in intraepithelial lymphocytes and plays a role in adhesion, migration, and lymphocyte homing (Kim et al., 2019). CD103 is highly expressed at mucosal sites, and it is very important in immune regulation (Annacker et al., 2005). The CD103 positive T cells play a vital role in antitumor immunity (Xiao et al., 2019). The expression of CD108 reflects the degree of tumor infiltration of CD8+ lymphocytes and can predict the prognosis in colorectal cancer (Hu et al., 2018). Therefore, T cells with highly expressed CD108 are involved in the immune reactions against antigens derived from tumors or pathogens. Additionally, the genotype-tissue expression (GTEx) database demonstrated that CD108 has an increased expression in lymph node, thus confirming our quantitative rules for indicating lymph node CD8 T cells.

The highly expressed CD357, also known as TNFRSF18 or GITR, can indicate the lymph node regulatory T cells based on our computational analysis. CD357 is a member of the TNF-receptor superfamily. This receptor protein plays a key role in immune regulation such as T-cell activation (van Beek et al., 2019). CD357 is overexpressed in regulatory T cells (Shimizu et al., 2002). CD357 is a co-activating molecule that is crucial in regulating T cell activation. This surface protein is highly expressed in murine and human regulatory T cells and is considered a regulatory T cell marker (Nocentini and Riccardi, 2005). These results strongly support the indicatory role of CD357 for recognizing regulatory T cells.

Ly6-high monocytes, which are also called inflammatory monocytes, express the high level of CCR2 and decreased CX3CR1 and play important roles in inflammation. Our decision rules showed that surface protein CD49d and gene CEBPB required a relatively high expression to indicate ly6-high monocytes. CD49d, which is also called ITGA4, belongs to the intergrin alpha chain family and may play a role in cell motility and migration. The regulation of ITGA4 is associated with inflammatory diseases (Gerecke et al., 2015; Zundler et al., 2017). Genetic study has reported that ITGA4 can influence the monocyte–lymphocyte ratio (Maugeri et al., 2011). Hence, CD49d is very critical for indicating ly6-high monocytes. As for gene CEBPB, the activity of its protein product is important in immune and inflammatory responses (Fields and Ghorpade, 2012). Bone marrow chimera experiments show that the deletion of CEBPB remarkably decrease ly6c monocytes, confirming the importance of CEBPB in monocytes (Tamura et al., 2017). Therefore, the criteria involving CD49d and CEBPB were validated for indicating ly6-high monocytes.

Results further show that a high expression of surface protein CD43 is required to indicate the spleen NK cells. CD43 is a highly sialylated glycoprotein that functions in antigen-specific activation of various immune cells. Despite the wide distribution of CD43 in monocytes, lymphocytes, and thymocytes, it is specifically expressed on human NK cells (McCann et al., 2003). The different sialylated forms of CD43 induce distinct functional roles of NK cells by transducing activation signals (Aguado et al., 1999). Notably, immature NK cells, which are mainly found in lymph nodes, usually lack CD43 expression, while mature NK cells defined as highly expressed CD43 preferentially migrate to inflammatory sites (Zhang and Meadows, 2008). These findings confirmed our rules that highly expressed CD43 on the cell surface can indicate the spleen NK cells.

In summary, our decision rules proposed that combined-cell surface protein or gene expression can be used to distinguish immune cell subtypes in different tissues. It validated the markers of each immune cell subtype and helps in discovering some new expression patterns of immune cells in the spleen and lymph node.

## Conclusion

The present study analyzed the single-cell CITE-seq protein and gene expression data of spleen and lymph, which involved 24 cell types. Through a computational procedure, including several machine learning algorithms, some essential features, powerful classifiers and classification rules were accessed. The analysis on the essential features, corresponding to surface proteins and genes, and rules indicated that they were related to the identification of spleen and lymph node cell types, suggesting they can be latent biomarkers for cell type identification. Furthermore, the powerful classifiers can be tools to classify spleen and lymph node cells.

## Data availability statement

Publicly available datasets were analyzed in this study. This data can be found here: https://github.com/YosefLab/totalVI_reproducibility/blob/v0.3/data.

## Author contributions

TH and Y-DC designed the study. HL, SD, and SZ performed the experiments. DW, XZ, WG, and ZL analyzed the results. HL, DW, XZ, and SD wrote the manuscript. All authors contributed to the research and reviewed the manuscript.

## Supplementary material

Supplementary Table 1 mRMR feature list yielded by the mRMR method.

Supplementary Table 2 Performance of four classification algorithms under different feature subsets.

Supplementary Table 3 Classification rules extracted by decision tree.
